# Radiomics in PET/CT and HRCT for systemic sclerosis-associated interstitial lung disease: breakthroughs and future directions

**DOI:** 10.1590/0100-3984.2025.0021

**Published:** 2025-09-01

**Authors:** Andréa de Lima Bastos, Marcelo Mamede

**Affiliations:** 1 Departamento de Anatomia e Imagem, Faculdade de Medicina da Universidade Federal de Minas Gerais (UFMG), Belo Horizonte, MG, Brazil

**Keywords:** Radiomics, Scleroderma, systemic/diagnostic imaging, Lung diseases, interstitial, Connective tissue diseases/metabolism, Precision medicine, Radiômica, Escleroderma sistêmico/diagnóstico por imagem, Doenças pulmonares intersticiais, Doenças do tecido conjuntivo/metabolismo, Medicina de precisão

## Abstract

Systemic sclerosis (SSc) is a multifaceted autoimmune condition that leads to fibrosis in the skin and various internal organs, including the lungs. One of its most serious complications is interstitial lung disease (ILD), which has a profound impact on the prognosis and on patient quality of life. High-resolution computed tomography (HRCT) plays a critical role by offering detailed structural information, whereas positron-emission tomography/CT (PET/CT) provides a deeper understanding of disease activity by combining metabolic and anatomical data. Radiomics expands on those modalities, extracting subtle imaging features undetectable by visual analysis, thereby enabling superior diagnostic accuracy, staging, and prognostic accuracy. This review explores the current applications of radiomics in SSc-ILD, highlighting breakthroughs such as the integration of artificial intelligence for early ILD prediction and risk stratification. Studies have demonstrated that radiomics is efficacious in overcoming traditional diagnostic limitations, enhancing precision in identifying the patterns of usual interstitial pneumonia and monitoring disease progression. When applied to PET/CT, especially that using advanced tracers, radiomics can complement HRCT by identifying metabolic biomarkers of ILD activity, thus supporting personalized treatment strategies. Although radiomics holds significant transformative potential, its routine use in clinical practice still faces several obstacles, such as the need for standardization, validation, and consistency across institutions. Future efforts will be focused on combining radiomics with genetic and molecular data, developing artificial intelligence-driven longitudinal models, and adopting multimodal approaches to improve the management of SSc-ILD. These advances promise to drive a shift toward precision medicine, ultimately improving outcomes for patients with this complex disease.

## Abstract (pt)

A esclerose sistêmica (ES) é uma condição autoimune multifatorial que resulta em fibrose cutânea e de diversos órgãos internos, incluindo os pulmões. Uma de suas complicações mais graves é a doença pulmonar intersticial (DPI), que impacta significativamente o prognóstico e a qualidade de vida dos pacientes. A TCAR exerce papel fundamental ao fornecer informações anatômicas detalhadas, enquanto a PET/CT, por integrar dados metabólicos à informação anatômica, permite uma compreensão mais abrangente da atividade da doença. A radiômica expande as capacidades dessas modalidades ao extrair características de imagem sutis e imperceptíveis na análise visual convencional, proporcionando melhor acurácia diagnóstica, estratificação e potencial prognóstico ampliados. Esta revisão aborda as aplicações atuais da radiômica na DPI associada à ES, destacando avanços como a incorporação da inteligência artificial para predição precoce da DPI e estratificação de risco. Evidências crescentes demonstram a eficácia da radiômica em superar limitações diagnósticas tradicionais, aumentar a precisão na identificação de padrões de pneumonia intersticial usual e monitorar a progressão da doença. A radiômica aplicada à PET/CT, especialmente com o uso de traçadores avançados, pode complementar a TCAR ao fornecer biomarcadores metabólicos da atividade da DPI, contribuindo para estratégias terapêuticas personalizadas. Apesar do promissor potencial transformador da radiômica, sua adoção na prática clínica ainda enfrenta desafios, como a necessidade de padronização, validação e reprodutibilidade entre diferentes centros. Futuros esforços concentram-se na integração da radiômica com dados genéticos e moleculares, no desenvolvimento de modelos longitudinais baseados em inteligência artificial e na adoção de abordagens multimodais, visando aprimorar o manejo da DPI associada à ES. Esses avanços sustentam a transição rumo à medicina de precisão, com perspectivas de melhoria nos desfechos clínicos dos pacientes acometidos por essa enfermidade complexa.

## INTRODUCTION

Systemic sclerosis (SSc) is a complex autoimmune connective tissue disease (CTD) that is most prevalent in women between the third and fifth decade of life, characterized by fibrosis of the skin and internal organs, such as the lungs, and associated vascular abnormalities^(1,2)^. Cardiopulmonary disease is the leading cause of mortality in SSc; interstitial lung disease secondary to SSc (SSc-ILD) is a significant complication, affecting over 50% of patients. The presentations of SSc-ILD range from sub-clinical pulmonary involvement to progressive lung disease, resulting in severe respiratory complications that have a negative effect on patient prognosis and quality of life. Early detection of lung lesions is essential for effective management of SSc-ILD^(3,4)^.

Histological patterns of chronic interstitial pneumonia constitute the most frequently seen component of SSc-ILD and have a variable clinical course. One of the most common histopathological patterns observed in SSc-ILD is that of usual interstitial pneumonia (UIP), second only to nonspecific interstitial pneumonia^(2,3,5)^.

The diagnosis of SSc-ILD involves the use of pulmonary function tests and morphological analysis of the lung parenchyma through the use of noninvasive, radiation-based imaging techniques. Among such techniques, high-resolution computed tomography (HRCT) is particularly important, although ^18^F-fluorodeoxyglucose positron-emission tomography/CT (^18^F-FDG PET/CT) has emerged as a promising tool for assessing disease activity^(1,6)^.

Although HRCT is pivotal in diagnosing, classifying, and monitoring lung damage^(4,7)^, the visual analysis of HRCT images is subjective and limited to morphological assessment of the parenchyma, which limits its clinical utility^(8)^. Quantitative methods have been proposed to address that limitation, enabling HRCT to estimate the extent of fibrosis, predict the decline in lung function, assess the risk of death, and detect treatment effects more effectively^(8,9)^.

The hybrid imaging modality PET/CT combines the metabolic insights of PET—commonly using ^18^F-FDG as a tracer—with the anatomical details provided by CT. In SSc-ILD, PET/CT facilitates the visualization of metabolic activity correlated with inflammation and disease activity, offering metabolic data that complement the morphological findings of HRCT^(6)^. Newly developed tracers show promise for broadening the applications of PET/CT and combining its metabolic information with HRCT-derived anatomical details to provide deeper insights into pathological processes^(10,11)^.

**Figure 1:**
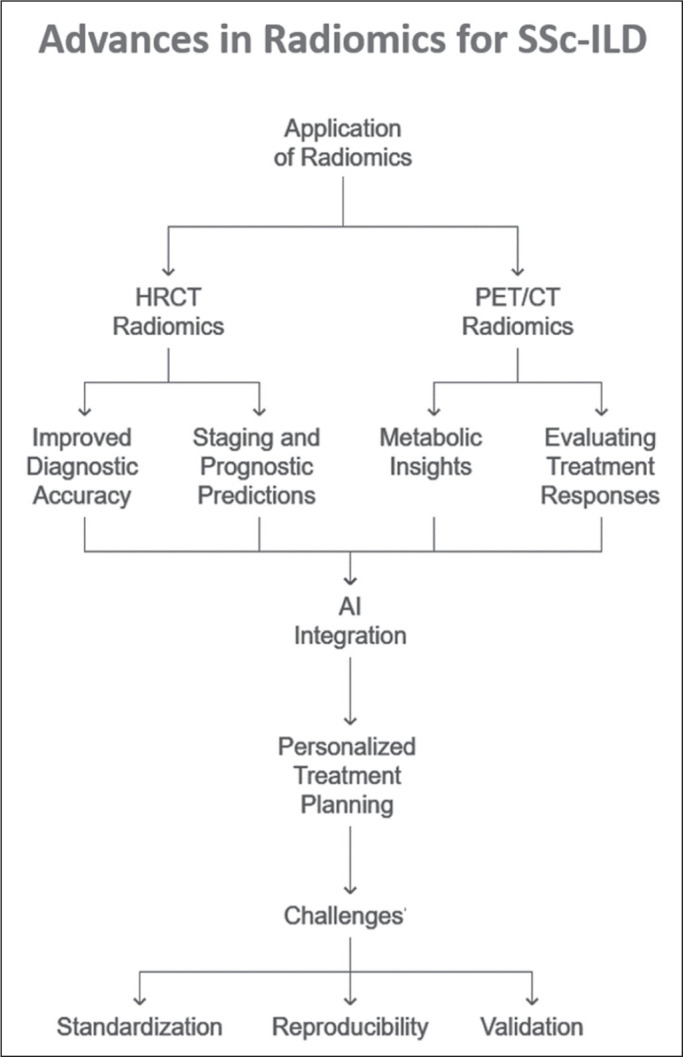
Conceptual framework illustrating the current and potential applications of radiomics in SSc-ILD. HRCT- and PET/CT-based radiomics contribute to diagnostic refinement, prognostic assessment, and evaluation of treatment responses. Integration with AI enables personalized treatment planning.

Technological advances have introduced artificial intelligence (AI), which has become a transformative force in medical imaging, enhancing the analysis and interpretation of HRCT and PET/CT scans^(12,13)^. Radiomics—a technique that is a sophisticated extension of computer-aided diagnostic systems—extracts and analyzes extensive quantitative features from medical images^(14)^. This technique enables the detection of subtle lung tissue changes that are invisible to the human eye, making it a powerful tool for characterizing ILD in SSc^(15)^. Radiomics has the potential to enhance diagnostic accuracy and identify novel imaging biomarkers, thereby improving clinical decision-making, especially when integrated with AI tools^(9)^. A framework illustrating the applications of radiomics in SSc-ILD is shown in Figure 1.

Despite the advances mentioned above, significant challenges remain in integrating radiomics and AI into routine clinical practice, including standardization, validation, and clearer interpretation of results. This review explores the current applications of radiomics in HRCT and PET/CT for SSc-ILD, highlighting recent advances and discussing future directions.

## STUDY DESIGN

This study was designed as a comprehensive review, summarizing current research and advances in the application of radiomics to HRCT and PET/CT images in patients with ILD secondary to SSc. The review includes an assessment of radiomics, its application in medical imaging, and the outcomes associated with its use. A comprehensive search of the literature was conducted via electronic databases such as PubMed/Medline, Web of Science, and Embase, to identify recent, relevant studies published between January 2020 and November 2024. The starting point of 2020 was specifically chosen because it marks the emergence of studies involving radiomics in ILD, providing a foundational framework for this evolving field. The search strategy employed the keywords “Artificial Intelligence”, “Tomography, X-Ray Computed”, “Scleroderma, Systemic”, “Lung Diseases”, “Positron Emission Tomography-Computed Tomography”, and “Interstitial Lung Disease”. Additional references were gathered through manual searches of the bibliographies of selected articles.

**Figure 2:**
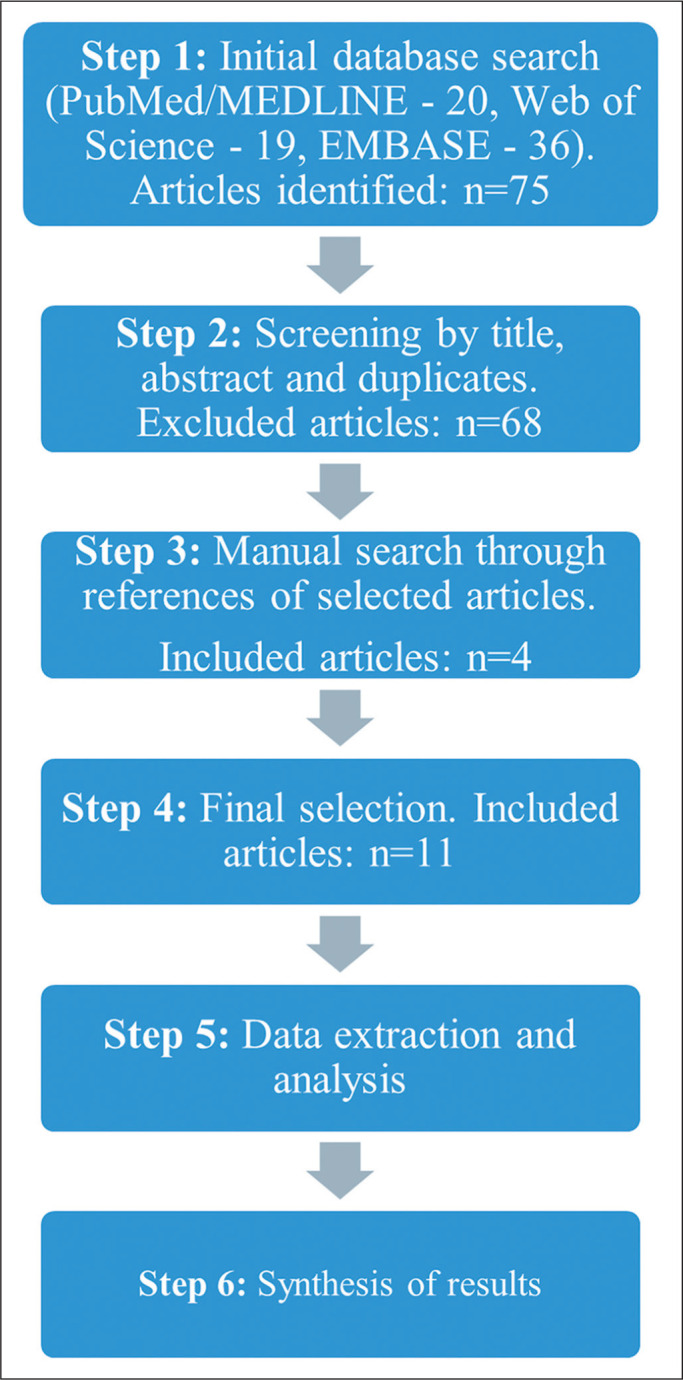
Flowchart of the article selection process.

The following inclusion criteria were applied: being an original study focusing on radiomics and AI application in the analysis of HRCT and PET/CT images in SSc-ILD; having been published in a peer-reviewed journal; presenting original research; and having been published in English. The article selection process is shown in Figure 2.

## RESULTS

**Figure 3:**
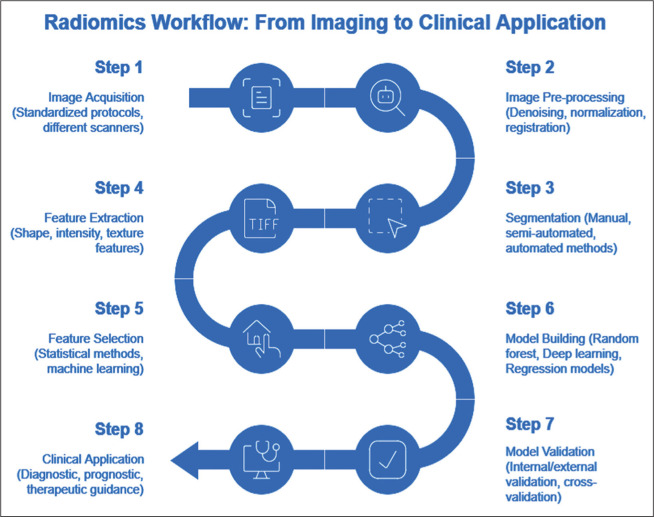
Overview of the radiomics workflow, illustrating the sequential steps from image acquisition to clinical application. Key stages include image preprocessing, segmentation, feature extraction and selection, model building using AI algorithms, and model validation.

A comprehensive review of the literature highlighted significant advances in the application of radiomics to HRCT and the emerging, albeit still preliminary, application of radiomics to PET/CT in SSc-ILD. Through a workflow that basically includes image preprocessing, segmentation, feature extraction/selection, model building using AI algorithms, and model validation (Figure 3), radiomics has shown promise in extracting detailed quantitative imaging features beyond those obtained with traditional visual methods. This tool can potentially improve diagnostic accuracy, staging, and prognostic accuracy. In PET/CT, radiomics holds the potential for expanded use as further research develops.

In radiomic workflows, AI algorithms such as regression models, random forest (RF) models, and deep learning (DL) models play complementary roles throughout the stages of feature selection, modeling, and interpretation^(16)^. Each of those models has specific advantages depending on the data structure, dimensionality, and clinical objectives. The RF model, an ensemble of decision trees, is widely employed for variable selection and classification because of its ability to handle numerous features and nonlinear interactions with robustness against overfitting, particularly in moderate-to-small datasets or when the number of predictors exceeds the number of observations^(17)^. However, the RF model can become computationally intensive and less interpretable when applied to high-dimensional data without prior feature reduction^(18)^.

The DL models, particularly convolutional neural networks, automatically extract hierarchical feature representations from raw imaging data, making them highly effective in image classification tasks and complex pattern recognition. These models are especially advantageous in recognizing subtle patterns undetectable by visual assessment, excelling in contexts involving large image datasets and requiring high diagnostic accuracy^(19)^. They excel in accurately identifying and classifying ILD subtypes, outperforming conventional methods and radiologists, particularly when provided with large, high-quality datasets^(20,21)^. Nevertheless, these models typically require large datasets and extensive computational resources, limiting their feasibility in smaller cohorts and early-stage studies^(22)^.

Regression models, including logistic and linear regression models, are typically applied in the final stages of radiomic analysis to construct quantitative predictive models. These models statistically relate selected radiomic features to specific clinical outcomes such as disease progression, treatment response, and risk stratification, by providing easily interpretable statistical associations^(23)^. However, their limitation is the assumption of linearity and difficulty in modeling complex, nonlinear interactions frequently present in clinical datasets^(18)^. The features and limitations of these algorithms are summarized in Table 1.

**Table 1:** Summary of the most common algorithms used in radiomic analysis of HRCT: key features and limitations.

Model	Key features	Limitations
RF	Robust with moderate data; handles many variables well; improves prediction accuracy^(18)^	High computational cost; reduced interpretability with many variables^(17)^
DL	Automatic feature extraction; high accuracy on large datasets; good for complex pattern recognition^(19,21)^	Needs large datasets to achieve optimal performance^(22)^
Regression	Predictive quantitative models; quantitative risk modeling; relates selected radiomic features to specific clinical outcomes^(23)^	Suitable for linear relationships; limited in complex scenarios^(18)^

Studies have shown that HRCT radiomics is an effective tool for stratifying patients with SSc-ILD on the basis of disease severity. It also correlates well with functional and biological markers of fibrosis. We find it interesting that radiomics models have been shown to surpass traditional visual scoring methods in predicting UIP patterns and staging ILD related to CTDs^(24)^. In addition, machine learning algorithms like RFs have proven to be highly accurate in detecting ILD early and monitoring its progression^(25)^.

Radiomics applied to PET/CT utilizing ^18^F-FDG uptake together with advanced tracers has been found to offer significant metabolic insights into inflammation and fibrotic activity. Semiquantitative, radiomics-based PET/CT models have shown potential in differentiating disease stages and evaluating treatment responses. Combining metabolic and anatomical data has improved the evaluation of disease heterogeneity and severity^(26,27)^.

The combination of radiomics and AI has improved diagnostic consistency and predictive modeling. The AI-based approaches that utilize radiomics features have been found to have superior sensitivity and specificity in comparison with traditional diagnostic methods, offering a pathway to personalized treatment planning.

Despite the advances mentioned above, there are still challenges in standardizing radiomics workflows, ensuring reproducibility, and validating results across multicenter studies. These findings underscore the transformative potential of radiomics in managing SSc-ILD, while emphasizing the importance of ongoing research and development to support clinical implementation. Table 2 provides an overview of the articles reviewed.

**Table 2:** Summary of studies on PET/CT and HRCT radiomics for SSc-ILD.

Reference	Year	Study location	N	Study design	Inclusion criteria	Methodology	Key findings
Anthony et al.^(28)^	2017*	Chicago, USA; Texas, USA	96	Retrospective	Esophageal cancer subjected to radiation therapy	Combined CT texture analysis with SUV from PET scans for RP prediction	Adding SUV data to CT radiomics significantly improved RP diagnosis, demonstrating multimodal radiomics potential
Salaffi et al.^(29)^	2020	Ancona, Italy	45	Cohort	SSc with follow-up HRCT	Comparison of quantitative methods (CaMs) versus visual scoring for ILD evolution assessment	CaMs outperformed conventional methods in detecting disease progression
Martini et al.^(30)^	2021	Zurich, Switzerland	60	Retrospective	SSc with GAP staging	Radiomics features extracted from HRCT for GAP staging.	Radiomics predicted GAP stage with high accuracy, surpassing visual analysis
Murdaca et al.^(25)^	2021	Genoa, Italy	38	Retrospective	SSc with HRCT and functional tests	Machine learning algorithms like random forest to identify early lung involvement	Random forest showed optimal performance for predicting early lung involvement
Refaee et al.^(31)^	2022	Maastricht, Netherlands; Wuhan, China	328	Observational	IPF diagnosis with available HRCT data	Radiomics model using HRCT for UIP detection	AUC > 0.96 for distinguishing UIP, reducing biopsy need
Schniering et al.^(15)^	2022	Zurich, Switzerland; Oslo, Norway	156	Prospective cohort	SSc diagnosis with ILD on HRCT	Radiomics features from HRCT for risk stratification	Radiomics effectively stratified risk and correlated with biological markers of fibrosis
Mei et al.^(20)^	2023	New York, USA	449	Retrospective	ILD on the initial chest CT scan	Deep learning model for ILD subtype classification	AI system showed high diagnostic and prognostic accuracy
Qin et al.^(24)^	2023	Shandong, China	245	Retrospective	CTD-ILD with HRCT data	Radiomics-based nomogram for GAP stage prediction	The nomogram achieved high accuracy in staging ILD (AUC > 0.85)
Joye et al.^(32)^	2024	Zurich, Switzerland	166	Comparative analysis	SSc with HRCT data	Radiomics comparison between slice-reduced and full-chest CT	Slice-reduced CT models slightly outperformed full-chest CT, minimizing radiation exposure
Zhao et al.^(33)^	2024	Hunan, China	58	Retrospective	Patients with SSc (ACR 1980 criteria) who underwent at least one lung HRCT.	AI-based analysis of HRCT to identify ILD lesion types and correlate them with clinical indicators and prognosis	AI successfully identified HRCT patterns and their progression; early ground-glass lesions were absent in cases with pulmonary hypertension
Smith et al.^(27)^	2024	Amsterdam, Netherlands	18	Exploratory pilot	Hodgkin lymphoma with bleomycin-induced ILD	PET/CT scans analyzed using radiomics and random forest classifier	Radiomics features (e.g., SUVmean, texture strength) identified and classified drug-induced ILD with 72% accuracy; predictive potential for early onset

* An older article (published in 2017) was included in this table because of its relevance in the PET-CT analysis.

GAP, gender-age-physiology; IPF, idiopathic pulmonary fibrosis; SUV, standardized uptake value; RP, radiation pneumonitis; CaMs, computed-aided methods.

## DISCUSSION

The heterogeneous, progressive nature of SSc-ILD poses substantial challenges in its diagnosis, staging, and monitoring. Radiomics provides innovative opportunities to enhance diagnostic precision and prognostic capabilities in SSc-ILD through advanced image quantification and feature extraction^(7,25)^. This discussion highlights the most recent advancements in PET/CT and HRCT radiomics, exploring their breakthroughs and potential future directions.

Several studies have highlighted the utility of HRCT-based radiomics in staging and monitoring SSc-ILD^(25^–^27,33)^. Notably, slice-reduced protocols have shown diagnostic performance comparable to that of full-chest CT, with the added benefit of reduced radiation exposure^(32)^. The quality of the features extracted from HRCT allows good detail and noninvasive follow-up of the disease without the subjective limitations of visual scoring.

The development of radiomics has provided great benefits for the evaluation of ILDs such as idiopathic pulmonary fibrosis. The radiomics-based model demonstrated excellent performance in distinguishing normal lungs from lungs with ILD—with an area under the curve (AUC) of 1.00, an accuracy of 99%, a sensitivity of 98%, and a specificity of 98%—and differentiating idiopathic pulmonary fibrosis with UIP patterns from non-idiopathic pulmonary fibrosis ILD, particularly when a typical UIP pattern is seen on HRCT—with an AUC of 0.96, an accuracy of 91%, a sensitivity of 88%, and a specificity of 94%^(31)^. According to Refaee et al.^(31)^, radiomics features extracted from HRCT, combined with clinical parameters, could support computer-aided decision-making, particularly in identifying and stratifying UIP patterns.

The performance of machine learning models in predicting early ILD in patients with SSc, on the basis of clinical and instrumental data, was explored by Murdaca et al.^(25)^. In their study, the RF model was trained to predict the Warrick score, a continuous measure of ILD severity. Among the algorithms tested by those authors, the RF model achieved the best performance, with a root mean square error of 0.810 and an R^2^ of 0.425 on the test set, demonstrating its suitability for ILD prediction^(25)^.

Martini et al.^(30)^ evaluated the potential of radiomics to detect ILD and assess its severity in patients with SSc, comparing it with that of visual analysis of HRCT images. The radiomics-based analysis demonstrated superior accuracy in predicting stages in SSc-ILD, with an AUC of 0.96, a sensitivity of 84%, and a specificity of 99%, compared with 0.86, 83%, and 74%, respectively, for the visual analysis^(30)^.

As shown by Salaffi et al.^(29)^, to assess ILD in SSc on HRCT, the quantitative features captured with computer-aided methods have also proven superior to those captured with visual analyses. In their study, the receiver operating characteristic curve showed that the performance of the computed-aided method to evaluate changes in HRCT—AUC of 0.951, with a standard error of 0.0287 (95% CI: 0.841–0.993) was better than was that of the visual analysis—AUC of 0.807, with a standard error of 0.0644 (95% CI: 0.662–0.909)—and the difference was significant (*p* = 0.0065).

One AI model was found to outperform human readers in identifying UIP and other ILD subtypes^(20)^. For UIP, the AI model achieved 82.4% sensitivity, surpassing the performance of senior radiologists and pulmonologists. That AI model enhances diagnostic accuracy and consistency, particularly for challenging ILD subtypes, and can incorporate longitudinal data for personalized survival predictions, guiding clinical management.

The integration of CT-based radiomics features with clinical factors for staging CTD-associated ILD can be used in order to predict ILD stages. These features have been shown to quantify subtle CT patterns that are not detectable by visual analysis, such as texture variations^(18)^. This methodology offers a quantitative, objective, reproducible method for staging CTD-associated ILD, reducing reliance on subjective visual assessments.

**Figure 4:**
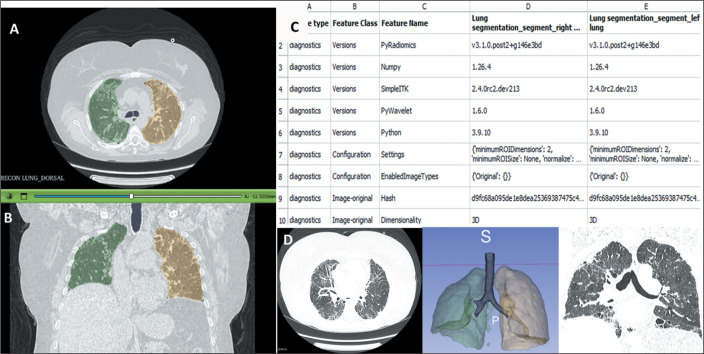
Radiomic analysis of HRCT using the software 3D Slicer, version 5.8.1 (www.slicer.org), in a 68-year-old female patient with SSc-ILD diagnosed approximately 30 years prior. **A,B:** Pulmonary segmentation obtained by lung CT analyzer. **C:** Radiomic analysis performed by radiomics module. **D:** HRCT slices (axial and coronal) and 3D reconstruction.

In a study analyzing the progression of lung lesions in patients with SSc^(33)^, AI was found to be able to identify distinct lesions, distribution patterns, and their progression, aiding in staging and prognostic evaluation (Figure 4). One important contribution was the ability to identify post-treatment changes in a lesion, such as a shift from consolidation to honeycombing.

Using ^18^F-FDG PET/CT to evaluate SSc-ILD offers unique insights into the metabolic activity associated with fibrotic changes. A semiquantitative analysis related to ^18^F-FDG PET/CT demonstrated that this modality can powerfully distinguish between ILD and normal lungs in patients with SSc^(11)^. However, it has mainly been applied in PET/CT imaging for the diagnosis, prognosis, and assessment of the treatment response in patients with cancer^(34)^.

Radiomics applied to ^18^F-FDG PET/CT represents a significant step forward in medical imaging, providing a quantitative, standardized approach to the diagnosis and management of lung disease. Despite technical and methodological challenges, ongoing research and standardization initiatives are paving the way for its broader application in clinical settings^(28,35)^.

The potential of ^18^F-FDG PET/CT radiomics and machine learning to identify and predict drug-induced ILD in patients with Hodgkin lymphoma treated with bleomycin was shown by Smith et al.^(27)^. Certain radiomics features, including texture strength and zone distance entropy, demonstrate potential for identifying drug-induced ILD in patients with Hodgkin lymphoma.

Future applications may involve integrating radiomics-derived metabolic gradients with machine learning algorithms for predictive modeling of disease trajectory and treatment response, leveraging metabolic data as a surrogate biomarker for early intervention^(28,36)^.

Despite advances in radiomics, challenges remain. Differences in scanner models, acquisition protocols, and segmentation methods can greatly impact the reproducibility of radiomic findings. Variability in scanner hardware, such as in detector types and reconstruction algorithms, together with inconsistencies in acquisition parameters, like tube current, tube voltage, and reconstruction kernels, introduce heterogeneity in image texture and noise characteristics^(37)^. These technical discrepancies can significantly alter the extracted radiomic features, leading to unreliable, irreproducible results, as well as limiting clinical utility and external validation. In addition, the method of image segmentation, whether manual, semi-automated, or fully automated, adds further variability^(37)^. Therefore, there is a clear need for harmonization and standardized reporting guidelines in radiomics research. Establishing robust, consensus-driven frameworks for acquisition, segmentation, feature extraction, and validation, such as the Radiomics Quality Score, is essential for achieving reproducible and clinically translatable radiomic biomarkers^(38)^. Furthermore, incorporating radiomics into routine clinical practice requires robust validation through multicenter studies and based on real-world evidence. By overcoming these barriers, radiomics could transform from a research tool into a key component in medical decision-making^(26,36)^.

The future of radiomics lies in its convergence with AI and multi-omics data, such as genomics, proteomics, and molecular profiles, paving the way for a new era of truly personalized medicine^(14,38)^. This radiogenomic approach enables the identification of imaging phenotypes linked to specific genetic mutations or pathways, allowing clinicians to better stratify patients, predict treatment responses, and tailor therapies accordingly^(39,40)^. The use of AI, particularly DL algorithms, plays a critical role by handling the complexity and volume of these multimodal datasets, uncovering patterns that would be undetectable through traditional analysis^(13)^. The integration of radiomics with molecular biomarkers is especially promising in oncology, in which combining imaging features with tumor genotypes can improve outcome prediction and treatment selection^(14,36)^.

In the future, the confluence of advanced imaging, machine learning, and personalized medicine could transform the care of patients with SSc-ILD. However, to turn this vision into reality, one would have to transcend the limitations of today and increase interdisciplinary cooperation with a view toward standardization and validation of these new methodologies.
